# Unilateral Common Carotid Artery Occlusion in Adult Mice with Streptozotocin Comorbidity Leads to Early Retinal Inflammation

**DOI:** 10.3390/ijms26094385

**Published:** 2025-05-05

**Authors:** Kate Gettinger, Deokho Lee, Kazuno Negishi, Toshihide Kurihara

**Affiliations:** 1 Laboratory of Photobiology, Keio University School of Medicine, Tokyo 160-8582, Japandeokho.lee@keio.jp (D.L.); 2 Department of Ophthalmology, Keio University School of Medicine, Tokyo 160-8582, Japan

**Keywords:** diabetes, diabetic retinopathy, streptozotocin, unilateral common carotid artery occlusion

## Abstract

Diabetic retinopathy (DR) is a leading cause of visual impairment. To better understand the pathology, clinically relevant experimental models are needed. Widely used DR models (especially streptozotocin (STZ)-induced) require extended timeframes to reach DR phenotype endpoints and lack ischemic phenotypes, which are in contrast to the human condition. Unilateral common carotid artery occlusion (UCCAO) could provide a retinal ischemic insult. We explored the pathologic synergistic effects of UCCAO in STZ mice. STZ (90 mg/kg) was injected intraperitoneally into adult C57BL/6 mice for three days. Four weeks later, right UCCAO was performed. One week after UCCAO, retinal samples were stained with isolectin B4 to analyze cellular and vascular changes. Retinal samples were obtained one day and one week after UCCAO and quantitative PCR (qPCR) were performed to observe inflammatory and ischemic responses. Only the STZ UCCAO group showed increased inflammatory cells. STZ UCCAO retina demonstrated a significant difference in capillary and large vessel size compared to other groups. At one day and one week, there was a change in mRNA expressions in inflammatory genes *Ccl2*, *Ccl12*, *Bnip3*, *Pdk1, Hsp25*, and *Vegfa* in the STZ UCCAO group compared to other groups. Our model can serve as an accelerated DR model for studying inflammatory vascular changes.

## 1. Introduction

One of the most frequent complications of diabetes mellitus is diabetic retinopathy (DR), which results from retinal microvascular complications [1]. DR is estimated to affect one-third of all people with diabetes and is the leading cause of preventable blindness in working-age adults [2]. Considering the estimate that over 783 million people will have diabetes by the year 2045 [3], it is imperative to find better treatments to prevent the inevitable rise of visual impairment due to DR.

Although ocular-immune-related diseases such as diabetic retinopathy have been subject to extensive study, there is still a gap in knowledge when considering the molecular mechanisms driving these [4]. A number of experimental animal models exist for studying both type 1 and type 2 diabetes, but each comes with its own strengths and limitations [5,6,7]. Several genetic rodent models include the Akita mouse, LEW.1AR1-iddm (LEW-IDDM) rat, and Nonobese Diabetic (NOD) mouse, but these models are not cost-effective, difficult to maintain, and do not completely replicate the human diabetic condition [5]. As far as chemically induced diabetic models, alloxan, streptozotocin, and cyclophosphamide exist as options [5]. Due to their cost-effectiveness and convenience, streptozotocin (STZ) mice are one of the most frequently used chemically-induced experimental models for studying diabetes [6].

In regard to studying DR, however, the STZ murine model has some notable limitations. DR phenotypes often take significant time to develop, with some findings taking close to a year to manifest, and most changes being nonischemic in nature [8,9,10,11]. Some neuroretinal changes, such as inner retinal thinning [12] and neural cell loss [13], have been demonstrated in STZ mice, but ischemic changes are more challenging to produce in STZ murine models. In Wistar rats with STZ-induced diabetes, for example, proliferative changes were not detected until 9 months after STZ induction [14]. It is hypothesized that DR onset in diabetic mice is delayed due to transiently activated protection from oxidative stress following diabetes induction [15]. While this could, in part, explain why mice and humans often demonstrate a delay between the onset of diabetes and the emergence of proliferative diabetic changes in the retina, from an experimental standpoint, where lab resources and time are frequently limited, it poses a challenge for studying these late-stage alterations. Overall, one of the largest downsides to the STZ model alone is that it rarely develops proliferative diabetic retinopathy changes [16].

The lack of consistent and reliable phenotypes that more closely resemble human clinical manifestations of DR, including proliferative diabetic retinopathy, create a challenge when attempting to understand the pathology and develop suitable treatments when using the STZ model. Instead, research scientists have used a model of oxygen-induced retinopathy [17] for studying retinal neovascularization, although there is no hyperglycemia in this model [16]. As such, it may not be a suitable replication of diabetic neovascularization changes. New models that can more accurately replicate human DR conditions within a shorter timeframe are desired.

Retinal ischemia is an important component of DR. It has proven especially difficult to fully understand, in part due to the lack of animal studies of retinal ischemia in diabetes [18]. In recent studies, a stable murine model of retinal ischemic stress has been suggested via unilateral common carotid artery occlusion (UCCAO) [19,20,21,22,23,24]. To date, acute retinal gliosis and neuronal degeneration have been detected in this model. UCCAO is a simple, reliable surgical technique to induce local ischemic stress in the central nervous system (including the retina) without experimental death. This carotid artery occlusion can be combined with other systemic metabolic stressors, including diabetes or obesity conditions [25].

In this study, we aimed to explore the pathological combined effects of UCCAO in adult STZ mice, establishing a new experimental model that can serve as an accelerated model for studying inflammatory vascular changes related to DR. We demonstrated that our accelerated model showed a significant increase in IB4+ staining inflammatory cells within the retina, a significant increase in mRNA expression of ischemia- and inflammation-related genes within the retina, a significant increase in average retinal large vessel diameter, and a significant decrease in average retinal capillary diameter.

## 2. Results

### 2.1. General Physiological Changes Observed in STZ-UCCAO Mice

**Figure 1:**
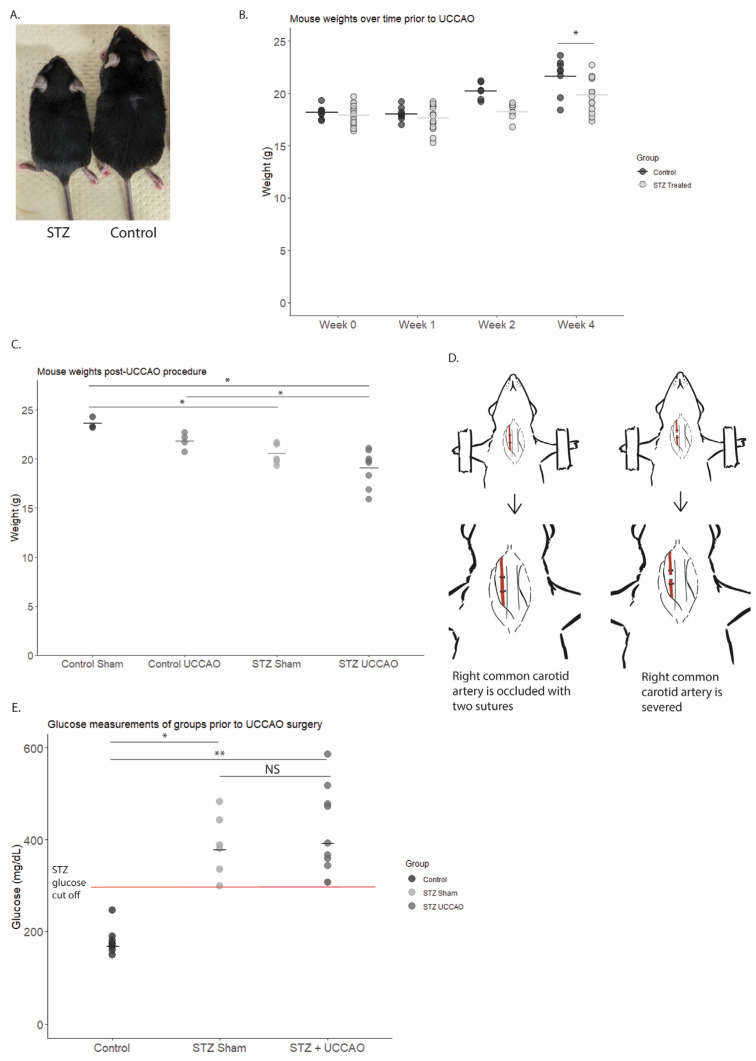
Streptozotocin (STZ) mouse characteristics pre- and post-unilateral common carotid artery occlusion (UCCAO). (**A**) Mouse body size comparison prior to UCCAO surgery (week 4). (**B**) Mean mouse body weights over time prior to UCCAO. Statistically significant decrease in mean body weight for STZ group (*n* = 18) compared to Control (*n* = 9) at week 4 (*p* < 0.01; unpaired Student’s *t*-test). (**C**) Mean mouse body weight post-UCCAO procedure. Significantly lower mean body weight of STZ UCCAO (*n* = 8) mice compared to Control Sham (*n* = 3) (*p* < 0.01; unpaired Student’s *t*-test). Significantly lower mean body weight of STZ Sham (*n* = 7) compared to Control Sham (*p* < 0.05). Significantly lower mean body weight of STZ UCCAO compared to Control UCCAO (*n* = 4) (*p* < 0.05; unpaired Student’s *t*-test.) (**D**) UCCAO procedure diagram. Right common carotid artery was isolated and tied with two 6-0 nylon sutures, then severed. (**E**) Mean mouse glucose measurements at week 5. Statistically significant difference between Control Sham (*n* = 7) and STZ Sham (*n* = 6) (*p* < 0.01) and Control Sham and STZ UCCAO (*n* = 10) (*p* < 0.01; unpaired Student’s *t*-test.) NS: Not significant. *,**: statistically significant (*p* < 0.05).

As expected due to the UCCAO procedure reported in the previous literature [20,26], mice with UCCAO, regardless of STZ status, demonstrated a right eyelid droop immediately following UCCAO (100%). Prior to the UCCAO procedure, as demonstrated in Figure 1A,B, STZ-treated mice demonstrated a statistically significant decrease in body weight compared to control mice. This is a reported characteristic of STZ mice [27,28]. After UCCAO, the resulting test groups were Control Sham (mice without STZ-induced diabetes and without UCCAO procedure), Control UCCAO (mice without STZ-induced diabetes but with UCCAO procedure), STZ Sham (mice with STZ-induced diabetes but without UCCAO procedure), and STZ UCCAO (mice with both STZ-induced diabetes and UCCAO procedure.) Figure 1C depicts the weight changes for all the mice groups one week after the UCCAO procedure. The STZ UCCAO mice had significantly lower body weight than Control Sham mice. STZ Sham mice also had a significantly lower body weight when compared to Control Sham mice. Among the UCCAO mice, the STZ UCCAO mice had a significantly lower body weight compared to Control UCCAO mice. Blood glucose levels were examined among groups (Figure 1E). While STZ mice demonstrated a significant increase in blood glucose compared to control mice, there was no significant difference in blood glucose levels between STZ mice receiving UCCAO or those receiving sham procedure.

### 2.2. Inflammatory Cells Increase in STZ UCCAO Retinas

**Figure 2:**
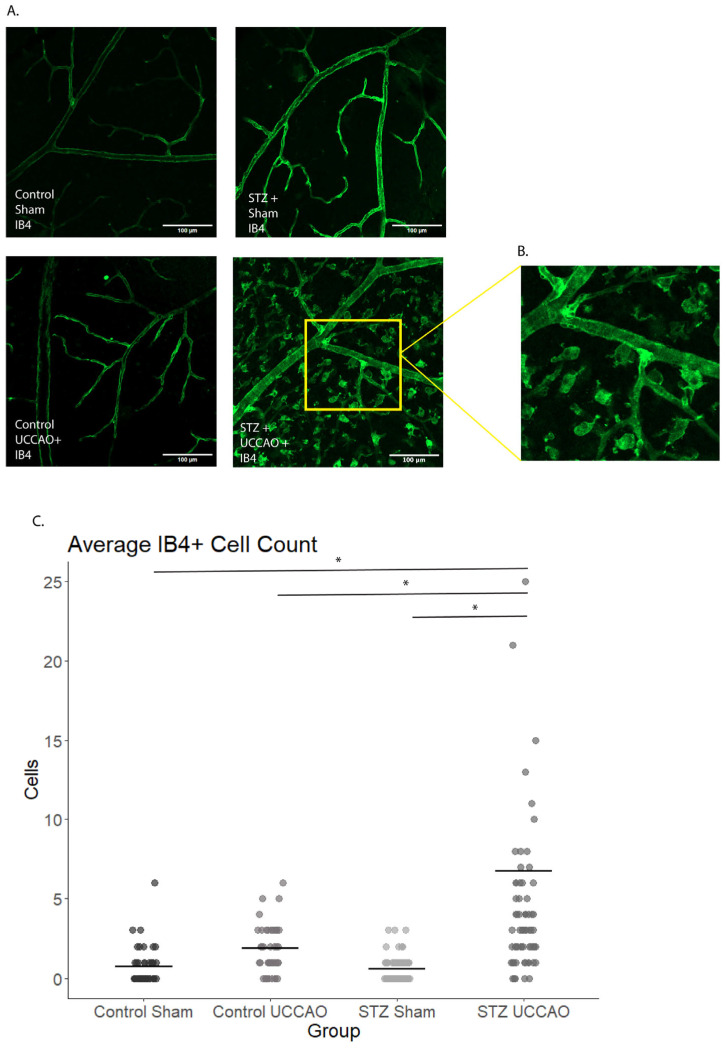
Comparison of IB4-stained inflammatory cells at 1 week post-UCCAO procedure. Cells were quantified using ImageJ software (Version 1.54k). Each quadrant of each retinal flat mount of right eyes was analyzed. (**A**) When comparing Control Sham, STZ Sham, Control UCCAO, and STZ UCCAO, STZ UCCAO demonstrated notably increased IB4+ cell count. (**B**) Enlarged detail of cells in STZ UCCAO retina. (**C**) Quantification of IB4+ cell count within Control Sham (*n* = 20), Control UCCAO (*n* = 22), STZ Sham (*n* = 18), and STZ UCCAO (*n* = 33) mice. Statistically significant increase in IB4+ staining cells per quadrant in STZ UCCAO group compared to Control Sham, Control UCCAO, and STZ Sham. * *p* < 0.05. Kruskal–Wallis test with pairwise Wilcox post hoc test. Graphs are depicted as the mean with each dot representing a sample value. Scale bar: 100 µm.

One week after UCCAO, there was a notable increase in the number of IB4-positive staining cells in the STZ UCCAO retina (Figure 2A–C). As seen in enlarged images, various morphologic inflammatory cells were detected in STZ UCCAO retina (Figure 2B).

### 2.3. STZ UCCAO Retinas Demonstrate Vascular Changes Not Described in STZ-Only Mice

**Figure 3:**
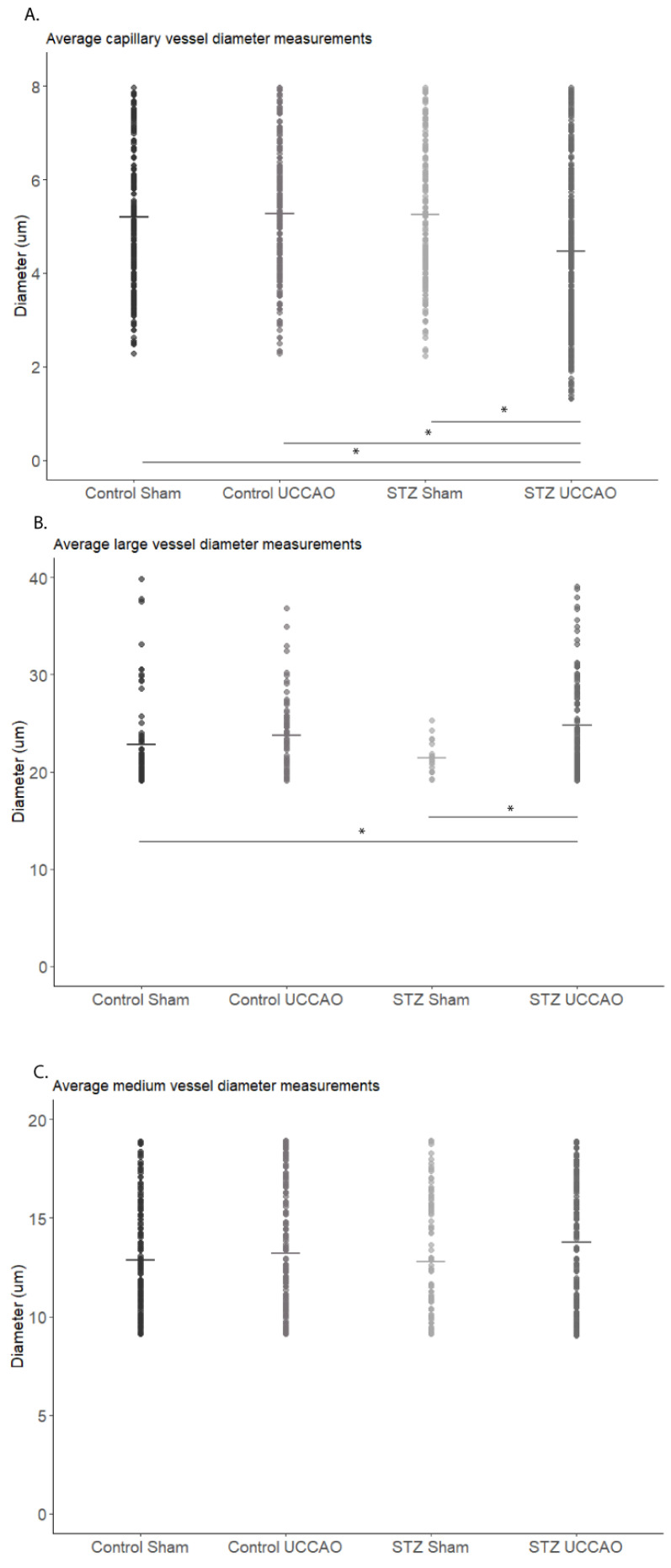
Average vessel diameter analysis for capillaries, medium, and large caliber vessels and average vessel area measurements. (**A**) Statistically significant decrease in average capillary vessel diameter in STZ UCCAO group (*n* = 50) compared to Control Sham (*n* = 30), Control UCCAO (*n* = 35), and STZ Sham (*n* = 19). (**B**) Statistically significant increase in average large vessel diameter for STZ UCCAO group compared to Control Sham and STZ Sham. (**C**) No significant differences in average medium vessel diameters between groups. * *p* < 0.05. Kruskal–Wallis test with Wilcox post hoc test. Graphs are depicted as the mean, with each dot representing a sample value.

When considering the vascular area for Control Sham, Control UCCAO, STZ Sham, and STZ UCCAO retinas, there was no statistically significant difference between the groups (Supplementary Figure S1). However, when analyzing changes in vessel diameters between the groups, there were noted differences (Figure 3A–C). In the STZ UCCAO group, there was a statistically significant decrease in the average capillary vessel diameter compared to Control Sham, Control UCCAO, and STZ Sham (Figure 3A). There was also a statistically significant increase in the average large vessel diameter for the STZ UCCAO group compared to Control Sham and STZ Sham (Figure 3B). There was no statistically significant difference in average medium vessel diameters between the groups (Figure 3C).

### 2.4. Alterations in Hypoxia-Related Gene Expressions One Day Post UCCAO in STZ Mice

**Figure 4:**
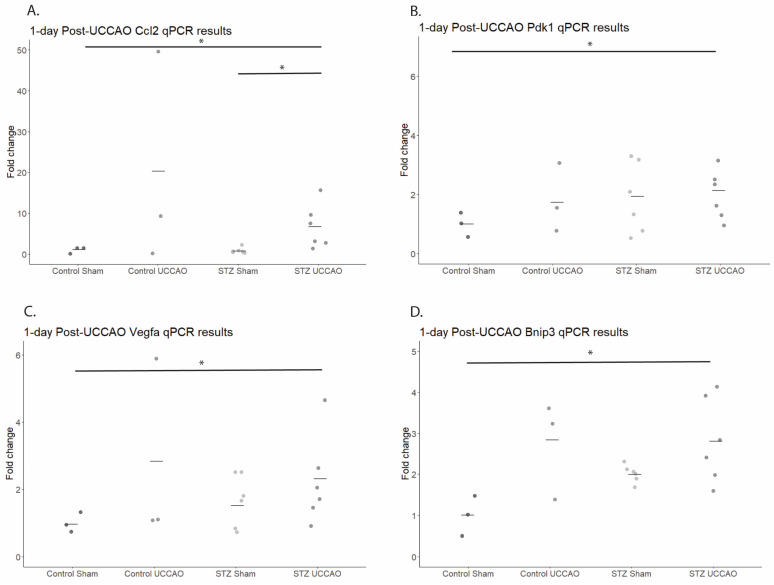
Alteration in gene expression levels in STZ UCCAO mice. (**A**) A quantitative analysis (*n* = 3–6 per group) showed that retinal *Ccl2* levels were significantly increased in the STZ UCCAO group compared to Control Sham and STZ Sham groups. (**B**) A quantitative analysis (*n* = 3–6 per group) showed that retinal *Pdk1* levels were significantly increased in the STZ UCCAO group compared to Control Sham group. (**C**) A quantitative analysis (*n* = 3–6 per group) showed that retinal *Vegfa* levels were significantly increased in the STZ UCCAO group compared to Control Sham group. (**D**) A quantitative analysis (*n* = 3–6 per group) showed that retinal *Bnip3* levels were significantly increased in the STZ UCCAO group compared to the Control Sham group. * *p* < 0.05. One-tailed Mann–Whitney test. Graphs are depicted as the mean, with each dot representing a sample value.

Previous studies have demonstrated upregulation of various hypoxia-related gene expressions 1 day after UCCAO surgery [20,29]. Therefore, we analyzed whether the addition of STZ to the model held any effect on these gene changes, and whether they persisted (Figure 4). A quantitative analysis demonstrated that retinal *Ccl2* levels significantly increased in the STZ UCCAO group compared to the Control Sham and STZ Sham groups. Retinal *Pdk1* levels were significantly increased in the STZ UCCAO group compared to the Control Sham. Retinal *Vegfa* levels were significantly increased in the STZ UCCAO group compared to the Control Sham group. In addition, retinal *Bnip3* levels were significantly increased in the STZ UCCAO group compared to the Control Sham group.

### 2.5. Alterations in Retinal-Hypoxia-Related Gene Expressions One Week Post UCCAO in STZ and Control Mice

**Figure 5:**
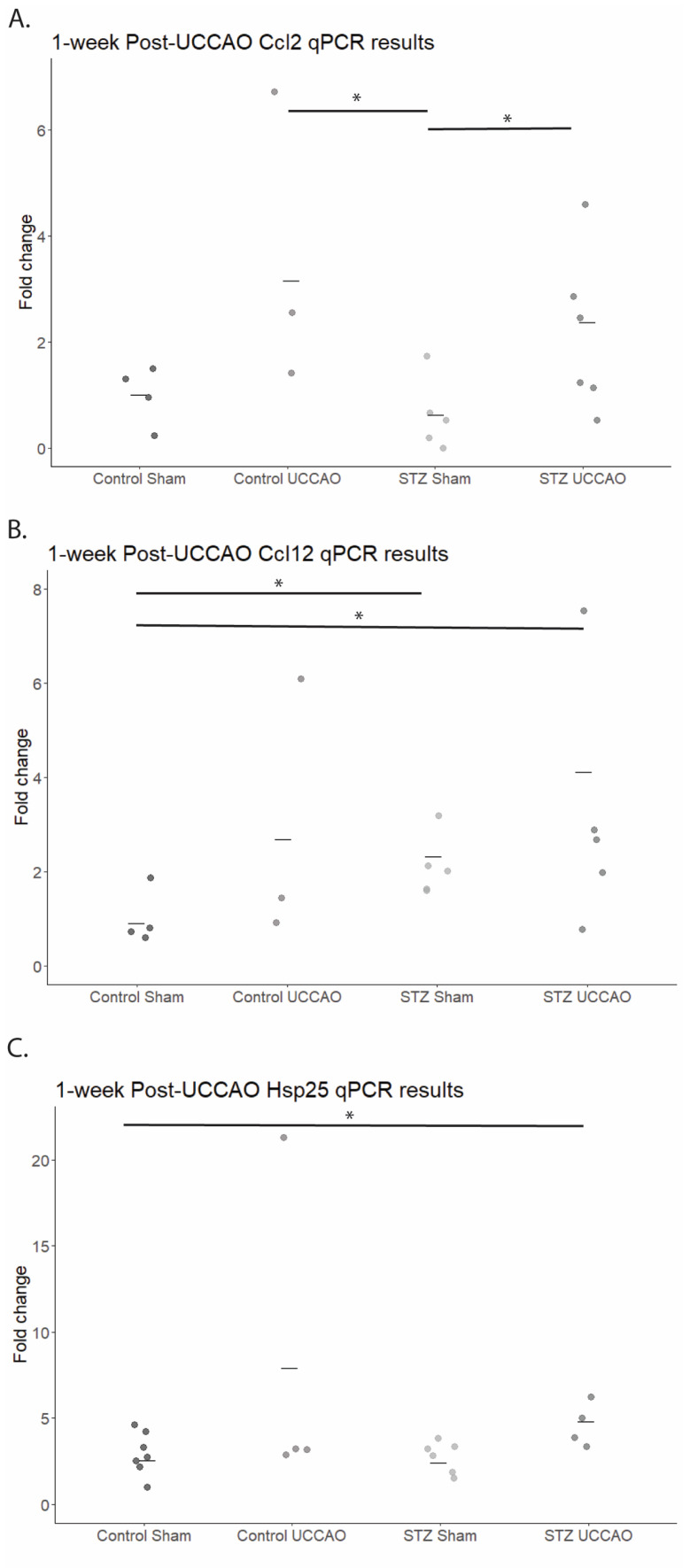
Alteration in gene expression levels in Control Sham, Control unilateral common carotid artery occlusion (UCCAO), Streptozotocin (STZ) Sham, and STZ UCCAO murine models one week after performing UCCAO. (**A**) A quantitative analysis (*n* = 3–6 per group) showed that retinal Ccl2 levels were significantly increased in the STZ UCCAO group compared to STZ Sham groups, and the Control UCCAO group levels were significantly increased compared to the STZ UCCAO group. (**B**) A quantitative analysis (*n* = 3–6 per group) showed that retinal Ccl12 levels were significantly increased in the STZ UCCAO group compared to the Control Sham group, and the Ccl12 levels in the STZ Sham group were significantly increased compared to the Control Sham group. (**C**) A quantitative analysis (*n* = 3–6 per group) showed that retinal Hsp25 levels were significantly increased in the STZ UCCAO group compared to the Control Sham group and the STZ Sham group. * *p* < 0.05. One-tailed Mann–Whitney test. Graphs are depicted as the mean, with each dot representing a sample value.

As previous studies additionally demonstrated that some hypoxia-related gene expressions remain altered up to 4 weeks after UCCAO [20], we also tested gene expression levels one week after UCCAO (Figure 5). A quantitative analysis demonstrated that retinal *Ccl2* levels were significantly increased in the STZ UCCAO group compared to the STZ Sham group. In addition, retinal *Ccl12* levels in STZ UCCAO retinas and STZ Sham retinas were significantly increased compared to the Control Sham retinas. Retinal *Hsp25* expression was also increased in the STZ UCCAO groups compared to Control Sham and STZ Sham.

### 2.6. STZ UCCAO Mice Demonstrate No Significant Changes in Refraction, Axial Length, or Choroidal Thickness Compared to Other Groups at 1 Week Post-UCCAO

To establish a more complete assessment of the STZ UCCAO phenotype, refractive measurements were taken, and optical coherence tomography (OCT) was performed to assess axial length and choroidal thickness (Supplementary Figure S2).

The refractometer results demonstrate no significant differences between the average refractive measurements of the groups (Supplementary Figure S2A). Similarly, there were no significant differences between either the axial length (Supplementary Figure S2B) or the choroidal thickness (Supplementary Figure S2C).

## 3. Discussion

In this study, we demonstrated that combining UCCAO with STZ-induced diabetes in mice can provide an accelerated model of DR that permits the study of inflammatory and ischemic vascular changes that are not consistently evident in STZ-only mice.

STZ is frequently used for animal studies of diabetic disease. STZ accumulates in the pancreatic β-cells and destroys them via free radicals and reactive oxygen species, simulating insulin-dependent diabetes [30]. In mice, a diabetogenic response is achieved through injection of STZ; however, there is some debate about the ideal dosage and timing of injections depending on what aspect of diabetes is being studied [31,32,33,34]. In addition, while STZ rodents have been shown to demonstrate inner retinal layer thinning [12] and retinal ganglion cell loss [13], notable complications such as neovascularization [14], oxidative stress [15], and other manifestations of DR are slow to develop or under debate [35]. As such, the mouse STZ model fails to consistently replicate the ischemic components of diabetic retinopathy that are seen in human conditions.

Based on previous studies, retinal vascular changes and ischemic changes in STZ rodent models are inconsistent and typically only visible after several months [14,36], occasionally taking up to 6 to 9 months to see signs such as microglia activation and neurodegeneration [37]. While its convenience and availability make STZ one of the most widely used animal models in DR research, it is mostly limited to studying the early phases of DR [5,36]. As its method of action is by destroying β-cells chemically, there is some instability due to the possibility of spontaneous β-cell regeneration and variations in mouse strain susceptibility [36]. This, in part, might explain why there are still disagreements about the appropriate dosage amount and injection timing to induce diabetes, as well as variety in the resulting phenotypes. As many of the desired late-stage DR findings in the mouse STZ model generally take months to develop and are inconsistent, this means there can be a significant amount of time and resources wasted on waiting for particular phenotypes to develop with limited success. Our desire was to combine STZ with another procedure to create a more consistent, predictable ischemic DR phenotype within a more reasonable timeframe.

UCCAO is a relatively new procedure for studying retinal ischemia, having been proven safer than the previously utilized bilateral common carotid artery occlusion procedures [20,26]. Retinal ischemia results from impeding the flow of blood to the eye, which leads to neurotransmitter disruption, oxidative stress, and cell death [38,39]. In humans, diabetes mellitus has been associated with carotid artery disease [40,41] and retinal vein occlusion [42], and, as such, the insult created by UCCAO could be considered to potentially mimic similar vascular stress seen in the human DR condition. This could mean that more clinically relevant interpretations may be drawn from a combined STZ UCCAO model, as the mechanism is not wholly dissimilar to human manifestations.

Previous studies of UCCAO mouse models have indicated the upregulation of inflammatory chemokines (*Ccl2* and *Ccl12*) [20,24,26] as well as ischemia-related genes (*Bnip3*, *Hsp25*, and *Vegfa*) [20,26,43] in the retina, and we found similar results with the STZ UCCAO mice. In this study, we demonstrated an increase in hypoxia-responsive genes in the STZ UCCAO retina compared to the Control Sham, with elevated levels of *Vegfa*, *Bnip3*, and *Pdk1.* The upregulation of these gene expression levels in the STZ UCCAO retinas also increased compared to STZ Sham retinas; however, the findings were not statistically significant. In addition, while these genes were significantly upregulated at 1 day post-UCCAO, this significance became only a trend when tested at one week post-UCCAO. This indicates that there is likely a rapid, immediate response of increased expression following surgery, but this expression is not sustained. Inflammatory signaling pathways have been implicated in diseases such as diabetes and age-related macular degeneration, but a comprehensive understanding of their role is still under investigation [44]. VEGFA is a multifunctional cytokine, demonstrated to play a notable role in vascular permeability and angiogenesis [45]. While our findings did not see significant changes in vascular density, there was a significant increase in IB4-positive cells within the STZ UCCAO retina, indicating a shift in permeability to permit migration of cells into the retina. The expression of inflammatory responsive gene *Ccl2* was also significantly upregulated in STZ UCCAO retinas compared to STZ Sham retinas, with a trend of *Ccl12* expression also being higher. CCL2, also known as monocyte chemoattractant protein-1, plays a role in the maintenance of monocyte and macrophage migration and infiltration [46,47]. In this model, *Ccl12* expression was slightly elevated at one day post-UCCAO, but it was not until one week post-UCCAO that a significant upregulation in expression became apparent, indicating that it may have a slow but steady increase following ischemic insult. CCL2, however, was significantly elevated at both time points. CCL2 has been associated with severe stages of DR and suggested as a potential target for future therapeutics [48]. Similarly to VEGFA, CCL2 has been indicated for its role in increased vascular permeability following acute stress [47]. This, taken along with the demonstrated increase in IB4 positive cells within the STZ UCCAO retina, and, to a lesser extent, the Control UCCAO retina, suggests that the STZ UCCAO model could be used to study ischemia-mediated DR inflammatory changes in the retina, shortening the time to reach DR phenotype. Macrophages have emerged relatively recently as a prominent player in various forms of ocular neovascularization [49]. IB4-positive cells might indicate activated microglia and monocytes, and their presence in the STZ UCCAO model can potentially reveal more insights into the role of ischemic stress in DR. This will be further studied.

BNIP3, or BCL12 19 kDa protein-interacting protein 3, has been associated with the HIF-1α hypoxia pathway, and is substantially upregulated in the inner retinal layer after ischemic insults [50,51]. Its upregulation within the STZ UCCAO retina further indicates the potential for this model to serve as a way to study ischemic complications in a diabetic condition within a controlled timeframe. PDK1, or pyruvate dehydrogenase kinase 1, is activated under hypoxic conditions and initiates the phosphorylation of pyruvate dehydrogenase, which then leads to its inactivation [52]. It has previously been indicated as a potential target for diabetic retinopathy treatment [53]; therefore, its upregulation in the STZ UCCAO retina is a promising finding that could permit more in-depth study. HSP25, a member of the inducible heat shock protein family, has been indicated as a protective response to ischemic insults [43] and has been shown to play a role in defense against retinal ganglion cell damage [54]. The elevated levels of *Hsp25* mRNA expression seen within the STZ UCCAO retina further suggest the successful addition of an ischemic component to the STZ diabetes model, as HSP25 is believed to act as a defensive mechanism in response to ischemia. We only saw significantly elevated *Hsp25* mRNA expression at one week following UCCAO, indicating that it may have a more delayed response to ischemic insult. Again, the upregulation of these gene expressions within the STZ UCCAO retina indicates it could be a model that more closely and consistently replicates later stages of DR in mice that previously have only been achieved over longer periods of time or through genetic manipulation [43,54].

We saw changes in the vessel diameter in both the capillary and large vessel category within the STZ UCCAO retina. Previous studies have indicated that rats with bilateral common carotid artery occlusion exhibit reduced capillary density as well as retinal thinning, and that narrowing of retinal arteries is commonly found in hypoperfusion retinopathies [55]. In contrast, some STZ mice studies have shown an increase in the number of retinal vessels [56,57]. Our study showed no significant difference in the density of vessels; however, we did observe significant changes in vessel diameter size. In STZ mice, reduced vessel diameters are more typically seen around 36 weeks [58], with other capillary vascular changes only evident at 6 months [59], indicating that our STZ UCCAO model may provide a vastly accelerated method to achieve these changes through the addition of the UCCAO procedure.

Previously, STZ-only mice have demonstrated thinned nerve fiber layer and ganglion cell layer via optical coherence tomography (OCT) [36,56], but changes in retinal pigment epithelium and choroidal thickness in UCCAO-only mice have shown more conflicting findings [22,24,26]. This leads to the question of whether the STZ UCCAO model might show any measurable changes via OCT within the 6-week timeframe, but at this time it still remains to be studied. In our study, we only examined retinal and choroidal changes at 1 week post-UCCAO, which was likely too early to see any significant changes. Previous studies suggest that the most notable thinning occurs closer to 20 weeks after diabetes induction in STZ mice [36]. However, we were curious to assess whether there was any evidence of edema within this short timeframe. Unfortunately, we did not see any edema as a result of the ischemic changes induced by the addition of UCCAO. Whether the addition of UCCAO accelerates the retinal thinning or induces notable edema is still unknown at this point, and testing at later timeframes, such as 3 or 5 weeks post-UCCAO, may be warranted. At the time of this research, our goal was to analyze rapid changes after administration of UCCAO to STZ mice. However, as UCCAO mice are stable and survive without unexpected sudden death [20], we believe that these mice could be tested at much later time points to see whether changes occur. To our knowledge, no studies have demonstrated significant refractive changes in either STZ or UCCAO mice, so our refractive findings were predictably unsignificant.

While we believe that the phenotype elicited by the combination of STZ and UCCAO is consistent overall, we do acknowledge that slight variation existed within the group. Some mice showed stronger phenotypes than others. However, as there were no outliers in the data, we do not believe that any of the significant findings were due to skewed data. It does beg the question, however, of why certain mice showed a stronger phenotype than others. All mice that successfully received the UCCAO procedure generally showed similar recovery, with no mice demonstrating significant behavioral changes, so superficially there did not appear to be any difference. We did not closely track blood glucose following UCCAO procedure, so one potential reason for variation is that mice that had persistently higher blood glucose levels following surgery demonstrated stronger phenotypes. Another potential cause for the variation might be due to individual differences in the UCCAO procedure itself. Due to the nature of the surgery, while all efforts were made to keep a uniform and consistent technique, there was some variation in detail. For example, the difference between the two occlusion sutures was not specified, mostly because we felt that it was unlikely that this would make a significant difference in our results. In addition, in some mice, the procedure took slightly longer to perform than others. Perhaps this extended surgery time created additional stress which resulted in a stronger ischemic phenotype. In the future, we intend to consider tracking these individual changes between mice and determining whether there is a correlation to a stronger phenotype. However, there is still the potential that these slight fluctuations in phenotype are just normal variations that we could expect to see in an experimental model of disease.

We believe that the STZ UCCAO mouse model of DR can serve as a useful tool for studying ischemic and inflammatory retinal changes that more closely resemble clinical conditions, but more study is needed to confirm whether there are any consistent functional visual changes to accompany these phenotypical changes. Previous studies have shown that STZ-only mice begin to show abnormalities in electroretinography (ERG) after 4 weeks [60,61], and UCCAO mice also demonstrate ERG dysfunction [26], and so we hypothesize that STZ UCCAO mice will likely show similar, or worsened, ERG findings, but it has not at this time been confirmed. STZ mice [62] and UCCAO mice [63] have also been shown to exhibit cognitive changes, which we have yet to measure in the combined STZ UCCAO mice. In addition, while we believe the STZ UCCAO to be a relatively low-skill procedure, it still requires more precision and expertise than a simple injection. There may be varying phenotypes and varying survival rates based on the skill of the experimenter, and careful consideration must be given to confirming the success of both the STZ procedure (through blood glucose verification) and the UCCAO procedure (through eyelid droop.) At this time, it is also unclear what the long-term prognosis and resulting phenotype of this model may be; thus, it remains a potential area to be investigated at a future date.

In summary, we attempted to develop a new, accelerated murine model of DR that demonstrates ischemic and inflammatory changes of later-stage DR through the combination of STZ-induced diabetes and the ischemic insult of UCCAO. We saw promising phenotype results in the upregulation of inflammatory and hypoxia-related genes, an increase in IB4-positive cell migration, and vascular diameter changes that all indicate that this model could be useful for studying ischemic vascular DR changes in mice within a relatively short timeframe compared to other models.

## 4. Materials and Methods

### 4.1. Animals

All animal experimental protocols were in accordance with the Ethics Committee on Animal Research of Keio University School of Medicine (Approval #16017/4 April 2025). In addition, procedures complied with the ARVO Statement for the Use of Animals in Ophthalmic and Vision Research in accordance with the Animal Research: Reporting in Vivo Experiments (ARRIVE) guidelines (https://nc3rs.org.uk/arrive-guidelines, accessed on 5 September 2024).

Five-week-old male C57BL/6 mice were obtained from CLEA Japan (Tokyo, Japan) and housed in a temperature-controlled environment under a 12 h light–dark cycle. The total number of mice used in all experiments was 165 mice. This sample size was chosen based on similar experiments and to confirm repeatability and reliability of the findings. All animals had free access to food and water. Mice were one-week adjusted to the environment after purchase before the experiment.

### 4.2. STZ-Induced DR

STZ-induction was conducted according to the schedule outlined in Supplementary Figure S3A–C. After randomizing, half of the mice received STZ (pH 4.5; 90 mg/kg) intraperitoneal injections for three consecutive days, while the other half were classified as the control group. Blood glucose was measured 7 days post-injection, and any mice with a blood glucose level greater than 300 mg/dL were categorized as STZ-induced diabetic mice. Body weight and glucose levels were periodically measured throughout the experimental timeframe for both control and STZ mice.

### 4.3. UCCAO Procedure

As depicted in Supplementary Figure S3A–C, UCCAO surgery was performed 4 weeks after STZ induction began. The control group and STZ group were randomized, with half of each group receiving UCCAO surgery. As demonstrated in Figure 1E, there was no statistically significant difference in blood glucose levels of STZ mice receiving UCCAO and those that did not receive UCCAO (sham group). The UCCAO procedure was performed as previously described [20,24,26]. In summary, mice were anesthetized using a combination of midazolam (40 μg/100 μL; Sandoz, Tokyo, Japan), medetomidine (7.5 μg/100 μL; Orion, Espoo, Finland), and butorphanol tartrate (50 μg/100 μL; Meiji Seika Pharma, Tokyo, Japan) (MMB) and placed on an operating table. A neck incision was made and the right common carotid artery (CCA) located. As depicted in Figure 1D, two 6-0 silk sutures were used to firmly occlude the right CCA. The right CCA was then severed between the two sutures, and the neck incision was closed with sutures. Mice were allowed to recover from the surgery in separate cages with a heating pad for a day. For those mice allotted to the sham group, the same procedure was performed except there was no occlusion. To confirm successful occlusion post-surgery, right eyelid drooping was observed by the naked eye as described by previous studies [26]. At the end of all experiments or if mice exhibited signs of impairment, such as lethargy, lack of food intake, or infection at surgical site, a combination of triple-strength MMB was administered and mice were euthanized under deep anesthesia.

### 4.4. Quantitative PCR (qPCR)

The qPCR procedure was performed at either 1 day post UCCAO or 1 week post UCCAO, as detailed in Supplementary Figure S3B,C. Eyes were enucleated, and total RNA was extracted from retinas via a commercial kit (RNeasy Plus Mini Kit, Qiagen, Venlo, The Netherlands), with concentrations determined using a spectrophotometer (NanoDrop 2000c, Thermo Scientific, Waltham, MA, USA). A commercial kit (the ReverTra Ace qPCR RT Master Mix with gDNA Remover, TOYOBO, Osaka, Japan) was used to convert total RNA to cDNA. Quantitative PCR was then performed using a PCR system (the Step One Plus Real-Time PCR system, Applied Biosystems, Waltham, MA, USA) and a SYBR Green master mix kit (THUNDERBIRD SYBR^®^ qPCR Mix, TOYOBO, Osaka, Japan). The fold differences were calculated by the ΔΔCT protocol. Primer sequences utilized within the study can be found listed in Table 1.

### 4.5. Immunohistochemistry (IHC)

One week after UCCAO was performed, IHC was performed as previously described elsewhere [26]. Briefly, mice were sacrificed and the eyeballs were enucleated and fixed with 4% paraformaldehyde (PFA). From the right eyes, the retinas were obtained, flat-mounted, and rinsed with phosphate-buffered saline (PBS) several times, then permeabilized with PBS containing 0.1% Triton X-100 and 0.1% BSA. The flat-mounted retinas were then probed with isolectin GS-IB4 from *Griffonia simplicifolia* (IB4) conjugated with Alexa Fluor 488 (Invitrogen, Carlsbad, CA, USA) and left to rest overnight at 4 °C. The flat-mounted retinas were rinsed again several times with PBS, then mounted on microscope slides with glass cover slips and examined using fluorescence microscopes (BZ-X800 Keyence, Itasca, IL, USA; LSM980 with Airyscan 2, Carl Zeiss, Jena, Germany).

For comparison of vascular changes via IB4 staining, mid-peripheral quadrants were analyzed for each retinal flat mount (Supplementary Figure S4A). Vascular area was measured for each quadrant in each right eye retinal flat mount using NIH ImageJ software (Version 1.54k, National Institutes of Health, Bethesda, MD, USA). Skeletonized versions of the vascular images were used to calculate differences in vascular area (Supplementary Figure S4B). Vessel diameter was also analyzed in each quadrant in each right eye retinal flat mount with NIH ImageJ. The two largest vessels and three random vessels’ diameters were measured by five raster lines per vessel, which were then averaged (Supplementary Figure S4C). Vessel diameters were then classified as capillaries, medium, or large vessels based on previous descriptions [64] and compared by group.

### 4.6. Ocular Biometric Characteristics Measurements

One week after UCCAO was performed, ocular measurements of refraction, axial length, and choroidal thickness were assessed by using an infrared photorefractor (Steinbeis Transfer Center, Graz, Austria) and an SD-OCT (Envisu R4310; Leica, Wetzlar, Germany) specifically designed for mice. Mice were administered 0.5% tropicamide and 0.5% phenylephrine eye drops prior to anesthesia to induce mydriasis. After confirming mydriasis, mice were anesthetized with MMB and then measurements were performed. Axial length was measured from the corneal vertex to the RPE layer. Choroidal thickness was measured and quantified from the OCT images using ImageJ, as described elsewhere [65].

### 4.7. Statistical Analysis

Testing with Shapiro–Wilk normality test for each sample confirmed the distribution of data, and appropriate testing was selected based on presence or absence of normality. Experimental values are presented as the mean ± standard deviation. All analyses were performed using R software v.4.3.2 (The R Foundation for Statistical Computing, Vienna, Austria). The statistical significance was set at *p* < 0.05.

## Figures and Tables

**Table 1:** Primer list.

Name	Direction	Sequence (5′-3′)	Accession Number
*Hprt*	Forward Reverse	TCAGTCAACGGGGGACATAAA GGGGCTGTACTGCTTAACCAG	NM_013556.2
*Ccl2*	Forward	CCCAATGAGTAGGCTGGAGA	NM_011333.3
	Reverse	TCTGGACCCATTCCTTCTTG	
*Ccl12*	Forward	GCTACAGGAGAATCACAAGCAGC	NM_011331.3
	Reverse	ACGTCTTATCCAAGTGGTTTATGG	
*Pdk1*	Forward	GGCGGCTTTGTGATTTGTAT	NM_172665.5
	Reverse	ACCTGAATCGGGGGATAAAC	
*Vegfa*	Forward	AAAGGCTTCAGTGTGGTCTGAGAG	NM_001025250.3
	Reverse	GGTTGGAACCGGCATCTTTATC	
*Bnip3*	Forward Reverse	GCTCCCAGACACCACAAGAT TGAGAGTAGCTGTGCGCTTC	NM_009760.4
*Hsp25*	Forward Reverse	CCTCTTCCCTATCCCCTGAG TTGGCTCCAGACTGTTCAGA	NM_013560.2

## Data Availability

The data are available from the authors upon reasonable request.

## Supplementary Materials

The following supporting information can be downloaded at: https://www.mdpi.com/article/10.3390/ijms26094385/s1.
