# Supplementary material for: Unilateral Common Carotid Artery Occlusion in Adult Mice with Streptozotocin Comorbidity Leads to Early Retinal Inflammation
Source: Int J Mol Sci. 2025 May 5;26(9):4385. doi: 10.3390/ijms26094385 (PMC12073014; doi:10.3390/ijms26094385)
Supplement: Supplementary file 1 [file ijms-26-04385-s001.zip › ijms-3578564-supplementary.pdf]

International Journal of
Molecular Sciences

Supplementary Materials:

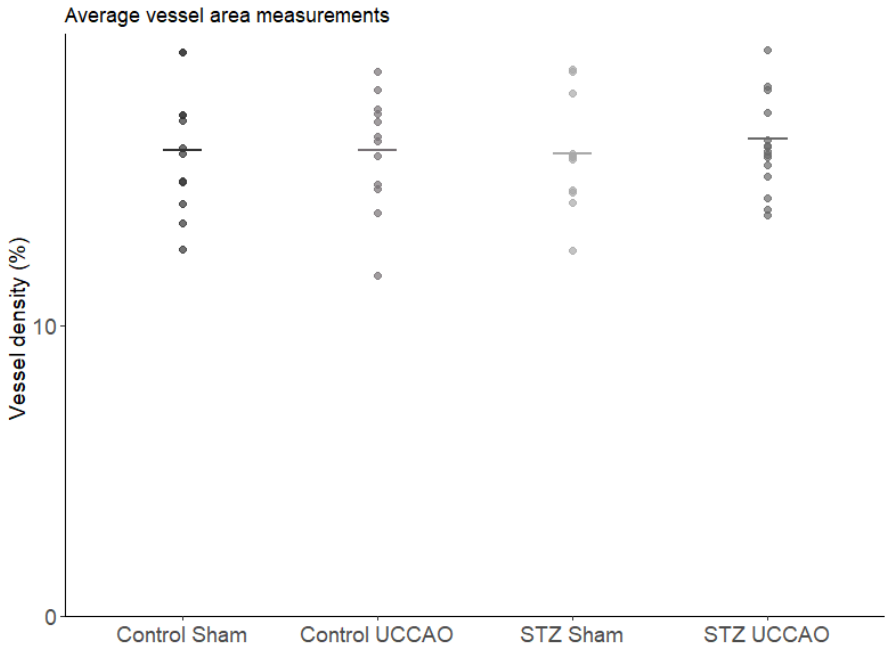

Figure S1. Average vessel area measurements. No statistically significant differences in average vessel area measurements between groups. Control sham *n*=12. Control UCCO *n*=12. STZ sham *n*=11. STZ UCCAO *n*=16.

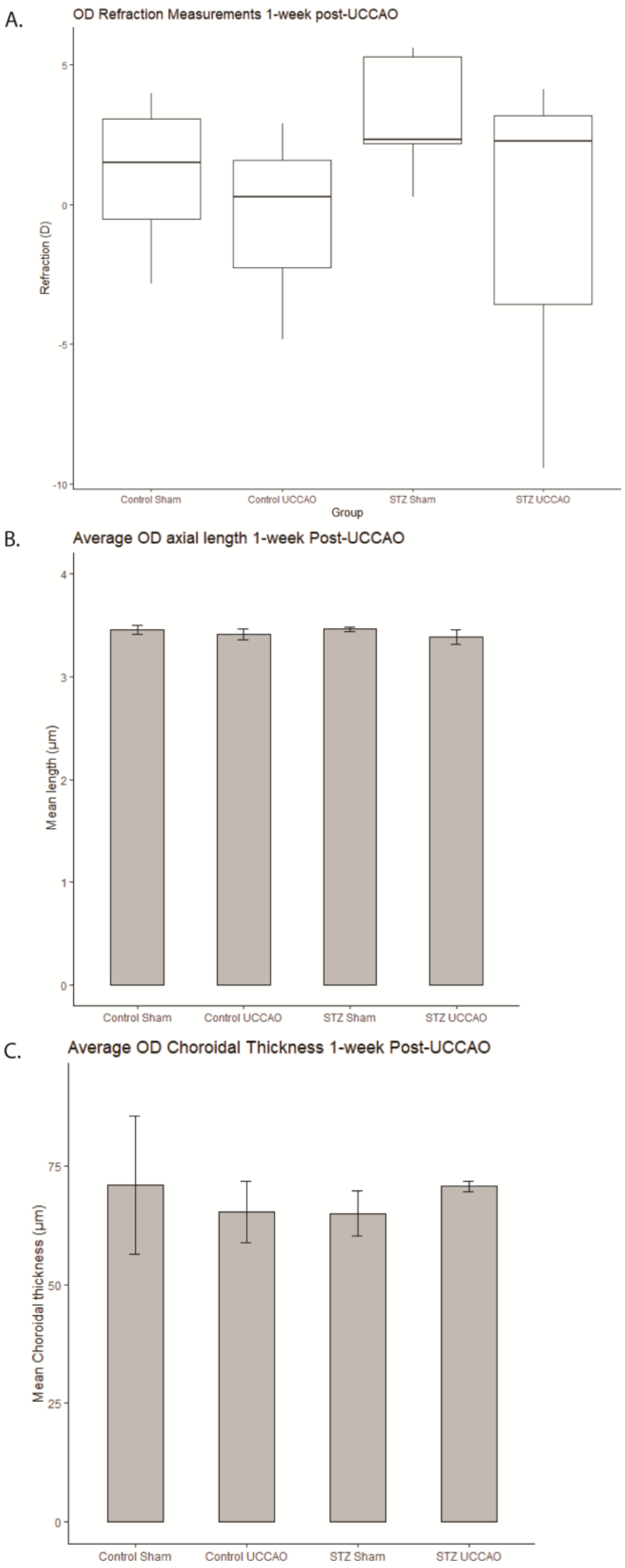

Figure S2. Refraction and optical coherence tomography (OCT) assessment results 1-week post-UCCAO procedure. **(A)** Average refractometer measurements demonstrated no significant differences between the test groups (*n*=3-6 per group.) **(B)** Average axial length did not significantly differ between groups at 1-week post-UCCAO (*n*=3-6 per group.) **(C)** Average choroidal thickness was not significantly different between experimental groups (*n*=3-6 per group.) D=diopter**.** *p < 0.05. Wilcoxon signed rank test. Graphs were depicted as the mean ± standard deviation.

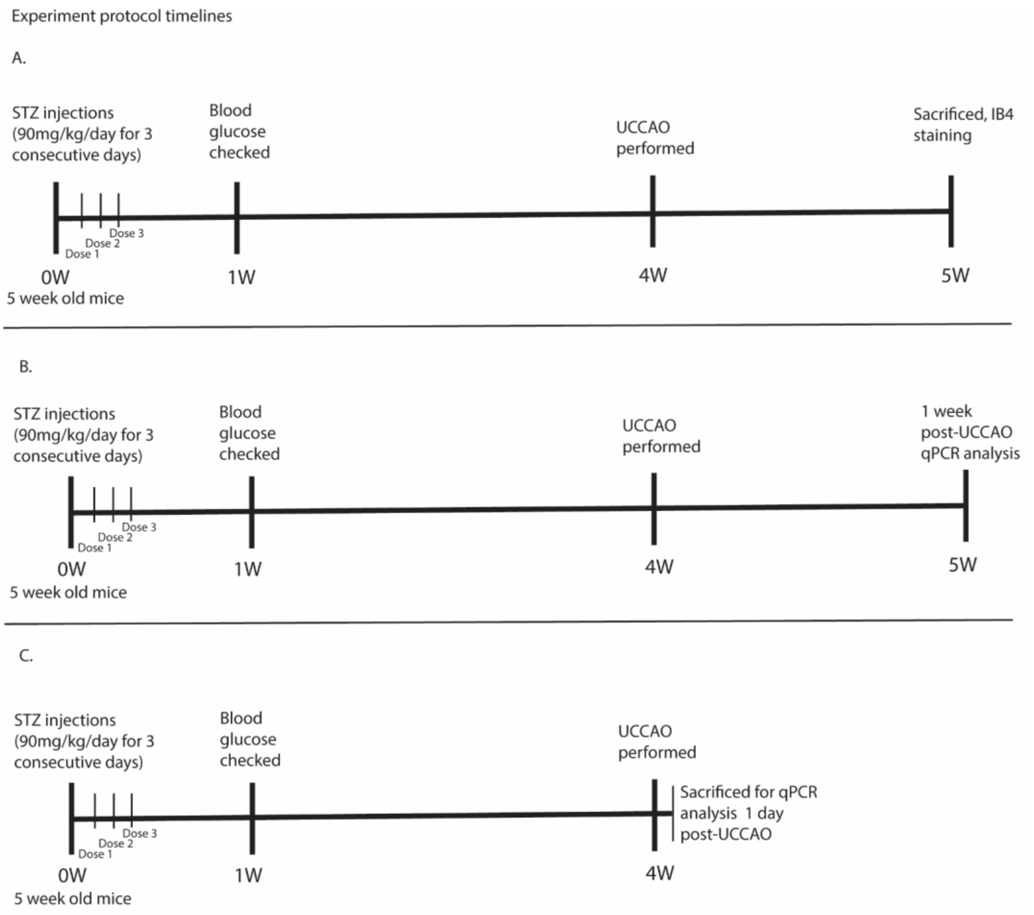

Figure S3. Experiment protocol timelines. (**A**) Baseline experiment timeline for isolectin-B4 (IB4) staining analysis. (**B**) Experiment timeline for one-week post-unilateral common carotid artery occlusion (UCCAO) qPCR analysis. (**C**) Experiment timeline for one-day post-UCCAO qPCR analysis. STZ: streptozotocin.

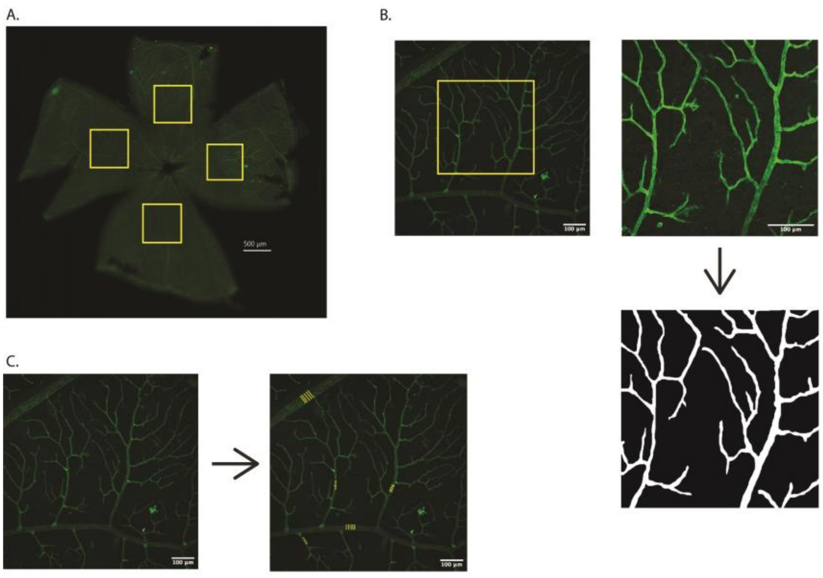

Figure S4. Vessel analysis methodology. (**A**) A representative image for retinal wholemount. Yellow squares indicate the approximate locations of mid-peripheral quadrants which were analyzed for each retinal flat mount. Scale bar: 500 µm. (**B**) Vascular area was measured in each quadrant of each retinal flat mount for right eyes using Image J software and a skeletonized version of vascular image. The green vasculature is the unmodified IB4 stained vasculature. The black and white image was created to isolate the green vasculature as white space, and the black representing nonvascular

space, so that Image J could easily calculate the difference in area. Scale bar: 100 µm (**C**) Vascular diameter was measured in each quadrant of each retinal flat mount for right eyes using Image J software (Version 1.54k).. The two largest vessels and three random vessels' diameters were measured by five raster lines per vessel, as illustrated. Scale bar: 100 µm.

Disclaimer/Publisher's Note: The statements, opinions and data contained in all publications are solely those of the individual author(s) and contributor(s) and not of MDPI and/or the editor(s). MDPI and/or the editor(s) disclaim responsibility for any injury to people or property resulting from any ideas, methods, instructions or products referred to in the content.
